# A Smart Waste Management Solution Geared towards Citizens

**DOI:** 10.3390/s20082380

**Published:** 2020-04-22

**Authors:** Kellow Pardini, Joel J.P.C. Rodrigues, Ousmane Diallo, Ashok Kumar Das, Victor Hugo C. de Albuquerque, Sergei A. Kozlov

**Affiliations:** 1 National Institute of Telecommunications (INATEL), Santa Rita do Sapucaí-MG 37540-000, Brazil; kellow.pardini@mtel.inatel.br; 2 Federal University of Piauí (UFPI), Teresina-PI 64049-550, Brazil; 3 Instituto de Telecomunicações, 6201-001 Covilhã, Portugal; 4 ITMO University, 197101 St. Petersburg, Russia; kozlov@mail.ifmo.ru; 5 Department of Informatics, University of Assane Seck, Ziguinchor 523, Senegal; odiallo@univ-zig.sn; 6 Center for Security, Theory and Algorithmic Research, International Institute of Information Technology, Hyderabad 500 032, India; iitkgp.akdas@gmail.com; 7 University of Fortaleza, Fortaleza–CE 60811-905, Brazil; victor.albuquerque@unifor.br

**Keywords:** Internet of things, IoT, smart cities, smart bin, waste management, waste disposal system

## Abstract

Global industry is undergoing major transformations with the genesis of a new paradigm known as the Internet of Things (IoT) with its underlying technologies. Many company leaders are investing more effort and money in transforming their services to capitalize on the benefits provided by the IoT. Thereby, the decision makers in public waste management do not want to be outdone, and it is challenging to provide an efficient and real-time waste management system. This paper proposes a solution (hardware, software, and communications) that aims to optimize waste management and include a citizen in the process. The system follows an IoT-based approach where the discarded waste from the smart bin is continuously monitored by sensors that inform the filling level of each compartment, in real-time. These data are stored and processed in an IoT middleware providing information for collection with optimized routes and generating important statistical data for monitoring the waste collection accurately in terms of resource management and the provided services for the community. Citizens can easily access information about the public waste bins through the Web or a mobile application. The creation of the real prototype of the smart container, the development of the waste management application and a real-scale experiment use case for evaluation, demonstration, and validation show that the proposed system can efficiently change the way people deal with their garbage and optimize economic and material resources.

## 1. Introduction

The latest developments in the Internet, with its underlying technologies, smart sensors and communication technologies, provide the possibility of connecting machines, devices, software, and objects communicating among them without human intervention, thereby paving the way for a new paradigm called the Internet of Things (IoT).

One of the main definitions of IoT from researchers, practitioners and businessmen is that IoT is a dynamic and global network infrastructure, in which intelligent things, subsystems and individual physical and virtual entities are identifiable, autonomous, and self-configurable [1].

Several efforts and research works have been dedicated to IoT technologies, such as Radio-Frequency Identification (RFID) technologies, sensors and actuators, wireless mobile communication technologies, embedded systems and cloud computing technologies. These advances allow IoT technologies to bridge the gap between ubiquitous network-based devices and technologies that monitor and collect information from physical world observations and provide new services and applications used to improve the living conditions of people in many areas. Thus, the IoT can deliver significant savings, improve utilization of a city’s assets, increase process efficiency, and add productivity by directly linking low-cost technologies. Some examples of these applications are smart cities, homes and offices, logistics and distribution systems, healthcare, surveillance and security, the supply chain, manufacturing industry, etc. [2,3,4,5].

In smart cities, the efficient management of waste is a crucial challenge for the environment that IoT tends to address [6]. Waste management covers all the activities necessary for monitoring the waste generated in a city, from its beginning, when citizens produce their waste, through collection, transportation, and arrival at its final accommodation, which can be the landfill, incineration, or recycling. It has been a significant challenge for cities around the world [7]. Thus, in the absence of an effective and efficient solid waste management program, waste generated by urban activities, both industrial and domestic, can result in health risks and harm the environment [8]. Understanding of the waste generated, the availability of resources and the environmental conditions of a given society is essential for the development of an appropriate waste management system. Solid waste is defined as materials that no longer interest the original owner and are discarded. Good examples are organic waste (including kitchen waste and leftovers from garden pruning), paper, glass, metals, plastics, fabrics, and wood. Solid waste management is associated not only with generation control but also with the disposal of solid waste in a way that follows the best principles of health, economy and other considerations as to the environmental attitudes developed by citizens.

Compared with developed countries, citizens of underdeveloped countries suffer most severely from the impact of unsustainably managed waste, In Brazil, for example, 80 thousand tons of solid waste are disposed of inadequately every day, according to the United Nations (UN) [9]. In these countries, garbage is often disposed of inappropriately in rivers, streets or even in open incineration; these practices have severe consequences for human health, safety and the environment. Improperly managed waste can serve as a rich source of disease and contribute to global climate change through the generation of greenhouse gases, and even promotes urban violence with the degradation of urban environments. Proper waste management is essential for the construction of sustainable and habitable cities but remains a challenge for many developing countries and cities. Effective waste management often becomes costly, compromising municipal budgets. Operating this essential municipal service requires an integrated system that is efficient and sustainable [10].

This work proposes an efficient and real-time waste management model for cities, focused on a citizen perspective. The proposed system includes sensor technologies where waste information is collected from the smart bin (things), in real-time, and then transmitted, through the Internet, to an online platform where citizens can access and check the availability of the compartments scattered around a city. A real prototype of the smart container was created, evaluated, demonstrated, and validated, and is ready-mapped in a real solution. The main contributions of this paper are the following:

- Proposal of a smart waste bin based on an IoT approach and the corresponding real prototype.
- Integration of the smart waste bin with an IoT middleware solution.
- Creation of a new mobile application and corresponding Web version offering a better interaction with residential users (waste generators).

The remainder of this paper is organized as follows. Section 2 presents related work on waste management, showing the most relevant solutions available in the literature. Section 3 describes the proposal of this study (a solution that aims to optimize the waste management process), including the creation of hardware, software, and integrated communication. The results of the performance study of the proposed system through a real prototype deployment are analyzed in Section 4. Finally, the conclusion and future works are identified in Section 5.

## 2. Related Work

Many research projects related to waste management are present in the literature. Saha et al. [11] brings a concept of intelligent disposal through a design that uses solar energy to feed the system and presence sensors for monitoring the amount of waste accumulated inside the enclosure. If necessary, the compartment can perform the compaction of the waste so that its volume can be reduced by up to 10 times, even before collection. Information about the fill level is sent via wireless communication to a cloud server where it is stored. The smart bin can act as a Wi-Fi hotspot, and it is easily adapted to any type of container, from small containers to large garbage containers such as underground containers. In turn, concessionaires access the system through a login and have access to data analysis, allowing fill level monitoring of smart bins, in real time, with notifications of need for collection through information that contains optimized routes for waste collection. This intelligent solution helps utilities reduce truck fleet, reduce fuel consumption, and maximize pick up time, minimizing operating costs by up to 80%.

Aiming to improve the work of cleaning operators to manage cleanliness issues in real time and to increase their productivity, the research work in [12] proposes a Smartbin system that identifies the fullness of litter bins. The proposed system collects data and transmits it through a wireless mesh network. In addition, Smartbin uses a duty cycle technique to reduce power consumption and to maximize operational time. This solution was tested in an outdoor environment for experiment validation, which shows that bin providers may well manage their litter bin utilization and the cleaning operators optimize their work.

A similar work is proposed in [13]. However, this smart garbage monitoring system can measure the garbage level in real time and alert the municipality when the bin is full based on the types of garbage. The proposed system uses ultrasonic sensors to measure the garbage level and an Advanced RISC Machines (ARM) microcontroller to control system operation, whereas everything is connected to ThingSpeak. The proposed system can show the status of four different types of garbage, such as domestic waste, paper, glass and plastic, through LCD and ThingSpeak, in real time, storing data for future use and analysis, such as prediction of the peak level of garbage bin fullness.

In [14], a specific proposal with a focus on the intelligent container was already presented. The authors propose an approach where monitoring not only occurs inside the compartment but also in the environment around it, in order to avoid waste disposal outside the container. The compartment is equipped with infrared sensors that play the role of detecting discarded garbage out of a bin, as well as measuring the compartment fill state. Infrared sensor signals that detect the garbage in the environment are delivered to an alarm system that is triggered to inform the person who disposed the garbage improperly; this alarm will cause people to dispose the garbage properly. For cases of waste accumulation around the container, the system has a mechanical lift consisting of a rack, electric engine, pinion, gear shaft, and chain pulley that are driven by a master controller and collect accumulated waste around the compartment. The grouping of the rotating mechanical axis together with the elevation ensures the common area around the waste-free compartment providing a clean, hygienic, and healthy environment for society. When the internal sensor detects the garbage limit level, the system automatically sends a message to the corresponding authorities, notifying them of the need for collection.

An intelligent collection system based on the level of residues present in the compartments and updated information on landfills is proposed in [15]. The system includes sensors installed in a compartment that determine the level of residues present internally through the distance measured from the cover and the beginning of the deposited garbage. For this, the authors use a sonar device like the HC-SR04. A battery-optimization process has been considered, which can be achieved through optimized waste detection rates (which can be done one or more times a day) in conjunction with a wireless transmission system (Wi-Fi is considered). This is a factor with strong influence on energy consumption that can raise the life of the device. The data obtained through the sensors installed in the trash can be transmitted to a MySQL database via the Internet and then, passed through optimization algorithms to calculate the best collection path. Associated with artificial-intelligence-based (AI) approaches, future waste levels can be predicted and properly associated with information from landfills, and a lower route of disposal can be determined. Every day, workers in the collection system update the paths on their navigation devices based on an essential feature of this system, which is to improve previous experience and to decide not only the status of the daily level of the compartments but also the foreseeable future state and other related factors, such as congestion, blockages, and parking area to receive the fleet at the end of a journey. Based on historical data on dumps and future projections it is possible to anticipate the occurrence of exhaustion of the landfills and, thus, plan new localities assuming less distance from the waste generation center.

Another proposal based on a garbage container solution is presented in [16]. The architecture of the solution is primarily based on an intelligent compartment that is responsible for updating the system with volume information, the type of content present in its interior, and the environment surrounding the place where the compartment is inserted. The enclosure is equipped with a range of sensors that enable detection and communication with the cloud and are managed through a microcontroller, such as Arduino Yun or a Latte Panda card, which receive the collected data, aggregate the data and transmits them to the cloud. This range of sensors is basically comprised by proximity sensors that provide neighborhood status data around the enclosure, such as information on restricted physical access for collection due to parked vehicles. There is a load cell that calculates the weight of the garbage available inside the compartment and updates the microcontroller. A humidity sensor is used to detect the level of dryness of the contents inside the compartment when it is not under usage for a long time, and the collection can be triggered when wet fragments are detected to avoid leakage. In addition, the compartment is equipped with a GPS that identifies your exact location. Also present is a lever-like drive key, which is used to detect open-lid physical events, generally due to over-filling of the tray. Complementing the solution, a mobile application is used by drivers of collection vehicles to determine the route and location of the bin within a collection schedule, and the application can be used by the public to control the disposal for a residence; QR code technology allows only registered users. The driver module inserted into the application ensures the implementation of dynamic routing by continuously monitoring the speed of the vehicle and its location. The cloud is the central processing unit of this system, which receives data pertaining to the management carried out by dumpsters. These data are aggregated and interspersed with weather conditions, rush time traffic, sporting events, and commemorative events with potential effects on the garbage truck route. The proposed system actively reacts to optimize waste collection routes.

The work presented in [17] also focuses in the context of waste management through smart bins. The presented model defends specific dumps applied to each type of waste and considers the following elements: wet/biodegradable paper; paper/clothing/wood; glass/metal; chemical/medical; and hazardous waste. In each compartment, there is a coupled GPS module that determines the exact location of the compartment, an infrared sensor to determine the compartment fill level, a gas sensor to detect harmful gases, a temperature and humidity sensor, and a sound sensor for noise pollution monitoring. All the sensors are managed by a microcontroller with a LoRa coupled communications module that is used to transmit the information obtained from the smart bin. A Linux-based gateway device (Raspberry pi) with LoRa module receives data from smart bins and, in the sequence, sends it to the cloud through a LAN/Wi-Fi connection using an MQTT message broker as the application layer protocol. The cloud layer includes data storage with a NoSQL database, event processing, and data analysis with alerts sent to the garbage trucks for collection when the boxes are full. These messages are received in an application that determines the best route for the truck to collect the waste.

For appropriate waste management in cities, the research work [18] proposes a framework of a Smart City Garbage Collection and Monitoring System. In this proposed, the smart bin is built on a microcontroller-based platform Raspberry Pi Uno board, which is interfaced with Global System for Mobile communication (GSM) modem and ultrasonic sensors and also a weight sensor, which is used for calculating the weight of the dustbins. Thus, the weight sensor is placed at the bottom of the dustbins, which will measure the weight of the dustbins, and the ultrasonic sensor is placed at the top of the dustbin, which will read the status of the dustbin. The Raspberry is programmed in such a way that when the dustbin is being filled, the remaining height from a threshold height will be displayed. When the waste achieves the limit level, an ultrasonic sensor will trigger a GSM modem, which will persistently caution the required expert until the trash in the dustbin is squashed.

The research work presented in [19] uses cloud technology and mobile app-based monitoring to provide a novel way of carrying out an integrated sensing system, which automates the solid waste management process. The proposed smart waste bin is based on ultrasonic sensors and various gas sensors, which provide it with the functionality to automatically sense the various odorous gases and the maximum limit of waste. Then, the required information is transmitted to the responsible authority. Another research work, presented in [20], proposes an IoT-based smart waste management system, which helps to survey the waste filling estimation in the dustbins and later transmit information through the Internet to a server for farthest point and treatments.

A commercial waste management solution, named Bigbelly, provides a public right-of-way platform to deliver smart waste management and host communications infrastructure is presented in [21]. Initially, Bigbelly was a solar-powered, rubbish-compacting bin, manufactured by U.S. company Bigbelly Solar for use in public spaces, including amusement parks, beaches, universities, retail properties, grocery industry, and food service operators. The bin was designed and originally manufactured in Needham, Massachusetts, by Seahorse Power, a company founded in 2003 with the goal of reducing fossil fuel consumption. Seahorse Power changed its name to BigBelly Solar due to the bin’s commercial success. Bigbelly is a Smart Waste Management, Smart City, and Internet of Things (IoT) industry leader and is recognized by the C40 Cities Climate Leadership Group as best practice. Deployed in over 50 countries, the Bigbelly solution claims to transform operations, drive efficiencies, increase productivity, and improve quality of life.

Similar to the previously presented solution, in [22], a commercial waste management live platform named smartbin is shown. The proposed platform is a complete SmartBin solution using a secure Web portal, at which IoT sensors report waste management information, in real time. Among the features of this solution, one can cite route optimization by generating efficient waste collection routes for only the containers requiring servicing; full management of container assets; and generating smart routes for drivers’ tablets or smartphones in garbage-emptying trucks.

Another commercial waste management solution named Bine is proposed in [23]. The proposed system includes IoT and big data technologies to well manage the public waste. This proposal includes functionalities such as waste recognition; sorting of waste types (glass, plastic, paper, metal, etc.); compaction of the waste to reduce the volume of the waste before collection; control of the fill level of garbage; and wireless communication toward a central processing system. This system supports monitoring, in real time, of the waste management and optimizes the logistics.

Much of the research work presented in this literary review deals with the management of solid waste with a focus only on the collection system, i.e., smart bins were developed for monitoring the waste discarded and generating information that can benefit the collection system through the positioning and the used volume of the compartments. This approach always aims to optimize routes for reducing collection time and costs regarding fuel, truck material, and human resources. There are also works that focus on a smaller transport route from the waste generation site to the landfill. Thus, data serves as information for predicting the exhaustion of landfill capacity and to search for new ones in areas around the waste-generating region, always keeping short routes from collection to disposal in the landfill, which keeps the focus on the collection system. Of course, it is assumed that the best approach for waste treatment should be recycling and reuse. The system proposed in this research gathers contributions from the described solutions proposed in the literature. However, to the best of the authors’ knowledge, few of them are focused on the perspective of the inhabitants that need to deposit the waste or present mobile-based solutions that can increase usability. Moreover, the available solutions have also shifted their focus to other aspects than IoT-based waste management integrated in an easy-to-use IoT middleware, which can be a huge contribution.

## 3. Proposal of the My Waste Management Solution for Citizens

The waste management system currently used in cities still follows an old and outdated model that no longer meets the needs of municipalities. It is inefficient and practiced through large fleets of collection trucks that travel daily long distances, often by unnecessary routes, where others are discovered, and with daily or weekly service schedules. These aspects bring unnecessary costs, waste of time and, more significantly, environmental damage, not only by the emission of gases from the burning of fossil fuel, which contributes to the greenhouse effect, but mainly by the contamination of soil and water resources due to poor waste management.

This paper proposes a solution that comprises hardware, software, and communication integrated into a solution that aims to optimize the management of the waste produced in cities through an approach that generates saving of the public money, contributes with the environment, and also encourages citizenship. 

In terms of research methodology, this study follows an approach based on a case study performed through a real deployment of the proposed solution. The created solution (a real prototype of the smart container and waste management app, integrated through the In.IoT middleware) is shown, demonstrated, and validated through real experimentation. The proposed solution is described below.

### 3.1. IoT Architecture Reference Model for the Waste Management System

To standardize the IoT segment or vertical, being supported by a reference architecture model is very important. Therefore, in the future, these waste management devices, which are called objects in IoT, can be connected and, therefore, the interoperability challenge is solved.

An architecture for IoT-technology-based applications is necessary when addressing factors such as scalability, interoperability, reliability, quality of service (QoS), etc. According to the authors of [2], there are several models and reference architectures available for IoT. Each research group or company describes its own, which often causes conflicts of ideas and makes the task of standardization more complex.

**Figure 1:**
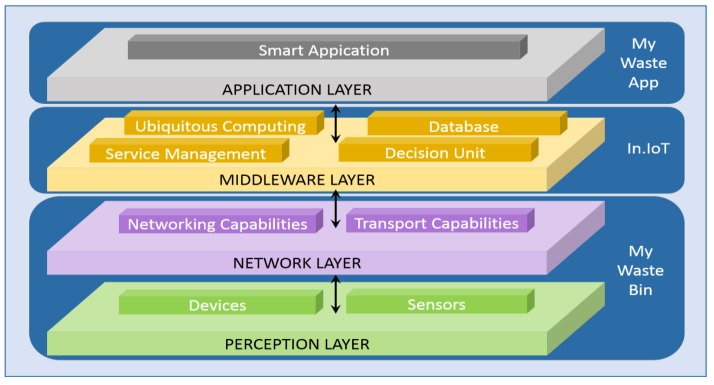
Layered architecture for the waste management system.

Many project models focus on a typical architecture based on needs analysis or on some layers that form a basic model of a reference architecture. The most basic approach only considers a three-layer architecture composed of application, network, and perception layers [24]. Figure 1 illustrates the basic layered architecture of the proposed solution.

These layers alternate according to the proposed model.

***Perception Layer:*** The IoT architecture perception layer is similar to the physical layer of the open systems interconnection (OSI) model, because it is based on the hardware level and has the responsibility of collecting physical information, processing and transferring it to the upper layers through secure channels. It applies technologies for the detection of parameters of physical characteristics through specific sensors, such as weight, temperature, humidity, etc. In addition, it performs the collection of object identification data, such as quick response codes (QR codes) and RFID.

***Network Layer:*** The network layer is responsible for transferring the measured information in the perception layer to the upper layers, where the processing systems are located, and uses ZigBee, Z-wire, GSM, UMTS, Wi-Fi, Infrared, 6LoWPAN. In addition to the basic assignments, the network layer also performs the cloud computing process and the data management process.

***Middleware Layer:*** The middleware layer is a layer of software or even a set of sublayers that work to interconnect components of the IoT that would not be possible to communicate otherwise, that is, as an interpreter. In addition to providing concurrency so that the application layer can interact with the layer of perception and ensure effective communication, it plays an important role in the development of new technologies.

***Application Layer:*** The application layer does not directly contribute to the construction of an IoT architecture, but it is in this layer where the various services are built that interface with users, that is, where the interpretation and availability of the information occurs.

### 3.2. Architecture of My Waste Management System

The system includes an applied solution where the compartments are monitored continuously by sensors, which inform, in real-time, the filling level of each one. These data are transferred to a storage and processing unit to serve as information, so that competent authorities can stipulate priority collection areas and collection paths with optimized routes and generate statistical data so the resources are employed adequately in regions with the highest demand for service. However, the main focus of the solution is to provide citizenship for residential users. Citizens can identify the compartments close to their home and know their level of usage in advance, via the Web or a mobile app. If the system recognizes unavailability at the nearest collection point, the user will be directed to discard his/her garbage at another available point and will receive the collection forecast from the previous bin, which allows the user to choose between a possible disposal at another location not so close, or even preserve the garbage at home so the disposal takes place at another time, after the municipal collection. 

**Figure 2:**
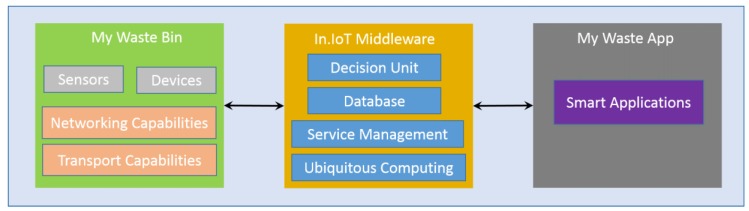
Illustration of the system architecture.

The My Waste Management system considers three main blocks, as shown in Figure 2. The first describes the smart bin, the second considers the IoT middleware integration, and the last block presents the user’s application.

Below, the whole system (hardware, software, and communication technologies) will be described, and it will be demonstrated also how it can be used to efficiently optimize waste management in cities. 

#### 3.2.1. My Waste Bin

The waste bin includes a container with a lid, and its enclosure is equipped with sensors such as the HC-SR04 module, an ultrasonic sensor responsible for measuring the level of waste filling present inside the compartment. This is significant within the solution, because through its operation it is possible to avoid the overflow of waste or excessive garbage deposit. The solution also includes a load cell module (load sensor) that measures the weight of the residues present in the compartment. It is characterized by a great importance within the system, since many residues have a small volume and significant mass. The load sensor is coupled to a specific driver, such as HX711, which amplifies the signal emitted by the load cell in addition to providing interconnection with the microcontroller.

The competence of these two modules is an aid to the HC-SR04, continually reporting the weight of the waste deposited in the compartment to contribute more comprehensive information on the residues present in the smart bin. A temperature and humidity sensor, such as the DHT11, is present in the solution to add relevant information about the environment where the enclosure is inserted. The air temperature and relative humidity are additional information added to assist the system user during the path for disposal of their waste. To enable smart bin tracking, a GPS module (model Neo-6M) was used to print geographic coordinates that represent the exact location of each bin. Through this location, the system will be able to inform users of the distances between them and the compartment closest to its location. 

The communication between the smart bin and the middleware is performed via a SIM900 GSM/General Packet Radio Service (GPRS) module. Through this module, communication using second-generation (2G) cellular technology is possible. It was decided to use this type of technology due to the extensive network infrastructure available and the low operating costs. Another important aspect is the access network coverage, as smart bins will be scattered throughout a city and often in the shadow area of mobile services. A technology that uses low-frequency radiation guarantees better penetration, preserving the proper functioning of the system. Finally, the system does not require large bandwidth for correct data transmission, so these aspects make the 2G technology entirely feasible for the proposed scope [25]. 

**Figure 3:**
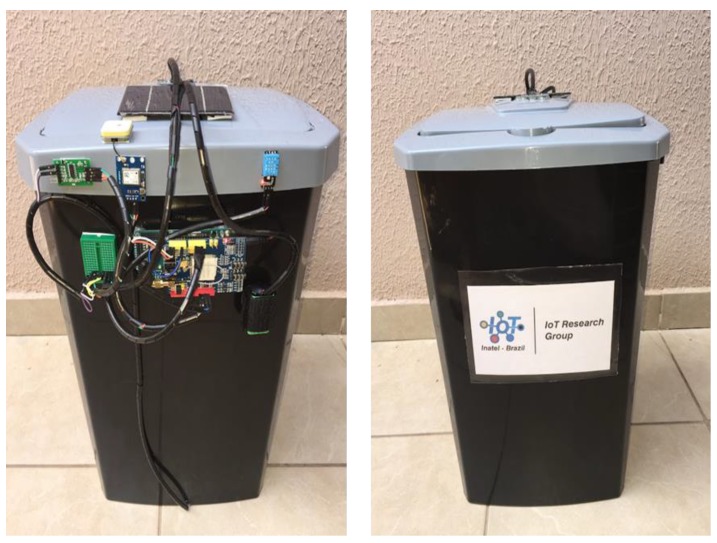
My Waste Bin prototype.

As a central processing system required to control the smart bin, an Arduino board [26] was used. This is based on a programmable physical circuit and an integrated development environment (IDE) used to write and upload code for a physical board. This open prototyping platform features a simple programming feature, ideal for IoT-based applications. The total power of the system is made by an external rechargeable battery coupled to a photovoltaic solar panel capable of recharging the cells during the daylight period. Figure 3 shows the image of the My Waste Bin prototype.

**Figure 4:**
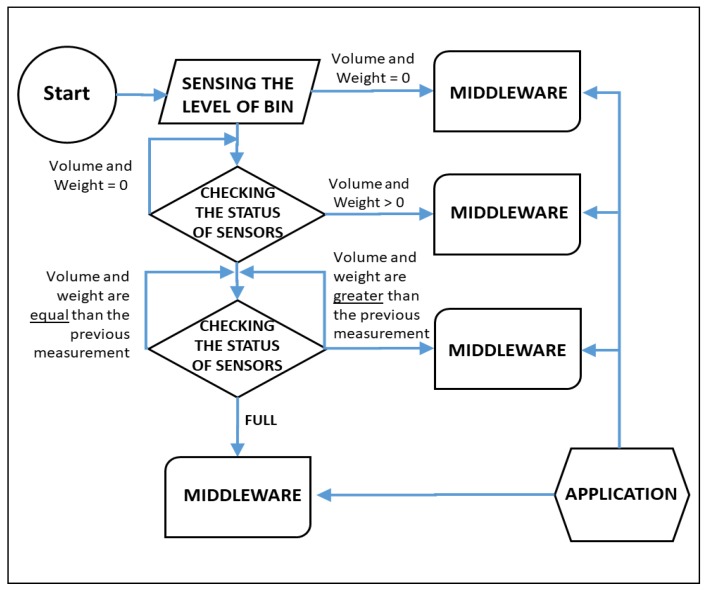
My Waste Bin Flowchart.

When initialized, the My Waste Bin system sends its geographical coordinates together with the date and time obtained through the satellite through the GPS module to the middleware. The weight and volume of the waste are transmitted initially as zeroes, along with the other data obtained through the sensors (temperature and the relative humidity of the air). These data are stored in the system and the compartment can already be found by the application as being able to receive waste. In the sketch of the Arduino [27], a timer instruction is inserted, and cyclically executes sensing to evaluate possible changes in the status usage of the compartment. Each time the filling level and volume is changed, a new transmission is executed, sending all the data obtained by the sensors. This sensing loop runs until the compartment attains its full fill level. From that moment, the application will report this compartment as full and the user (citizen) will receive new waste disposal options through other compartments available in the area. For the concessionaires, it is already a compartment eligible for collection. Figure 4 illustrates the working flow diagram of the smart My Waste Bin.

#### 3.2.2. IoT Middleware Integration

**Figure 5:**
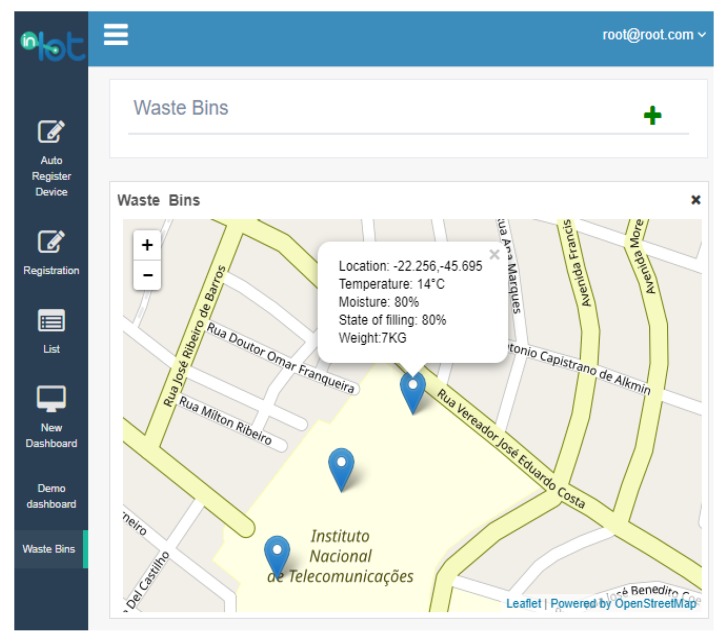
Image from the In.IoT middleware platform showing the location of smart bins.

Middleware is a software that connects base systems such as IoT devices to each other and third-party applications [28,29]. It works as a layer of translation, allowing communication and data management for distributed applications. Within the waste management system (and other IoT systems), the middleware plays an essential role because it receives all the data sent by the containers and stores them so that specific queries executed by the application are quickly and consistently accessible to the user. In this case, the middleware used was In.IoT [30,31], which also allows users to visualize the waste bin status in real-time via the Web. A screenshot of the In.IoT middleware platform, presenting the location of smart bins around the urban region where the solution is used, is shown in Figure 5.

#### 3.2.3. My Waste App

Over the years, mobile phones have evolved from simple devices that just enable communication between people to powerful application processing centers where any task is possible. Therefore, modern users would never use a smart waste management solution if not through their smartphone. With this in mind, the My Waste App was designed. It is a hybrid mobile application built with Ionic and is compatible with the Android and IOS mobile operating systems. The app queries data sent to *In.IoT* and allows users to identify and verify the status of nearby waste disposal sites. In addition, the user can also visualize predictions of disposal sites, allowing users to dispose of their waste after municipal collection.

Ionic [32] is an open source framework used for creating mobile applications for various existing platforms. It was chosen as a tool for the construction of My Waste App because it is free and presents a simple and easy interaction user interface, besides using technologies usually employed in Web solutions, which give the app a very professional aspect.

When starting to build an app it is very important to have a list of well-defined requirements; good requirements provide the development of a clearer system and come closer to achieving user satisfaction. A weak application of requirements can lead to the disuse of the app and, thus, ruin the entire productive chain of the system.

Given the importance of this aspect, My Waste App takes into consideration the following requirements: the user need, the requirement for business, and the desire to be met with the system. The residential user has a well-defined need, which is to dispose of their waste in a practical, fast way without harming the environment; the requirement of the business is to identify the waste generation sites, capture them correctly using appropriate compartments, and encourage user interaction with the system through updates of relevant information, such as the location and available capacity of the compartments, collection scheduling, and (of course) feedback from the users. The system aims to provide citizenship (show the users that each one is responsible for the waste generated until the time of disposal), provide a cleaner environment free of setbacks due to poor waste management, minimize waste collection system, and contribute to reducing the traffic of automotive vehicles.

The My Waste App application can be customized according to the profile of each user, with the possibility of a favorite container register, such as, for example, a container located closer to his/her residence, along with the days of a week and the time that he/she frequently uses to dispose of his/her waste. This way, the application can automatically check the status of the favorite container and pass usage information through a pop-up. If the compartment has a maximum fill level, the collection schedule is already displayed along with a second available bin option. The application can also be used through sporadic queries at times of discard, without the need to register favorites, so the system crosses the location of the user’s smartphone with the containers positioning and offers the option that is most feasible for the moment. The purpose of the system is to help users practice proper disposal of their waste by presenting waste disposal options available to receive their waste even before the user leaves the house. If no option is feasible at the time, the user can consult a collection schedule and reserve their waste at home for disposal at a more appropriate time.

**Figure 6:**
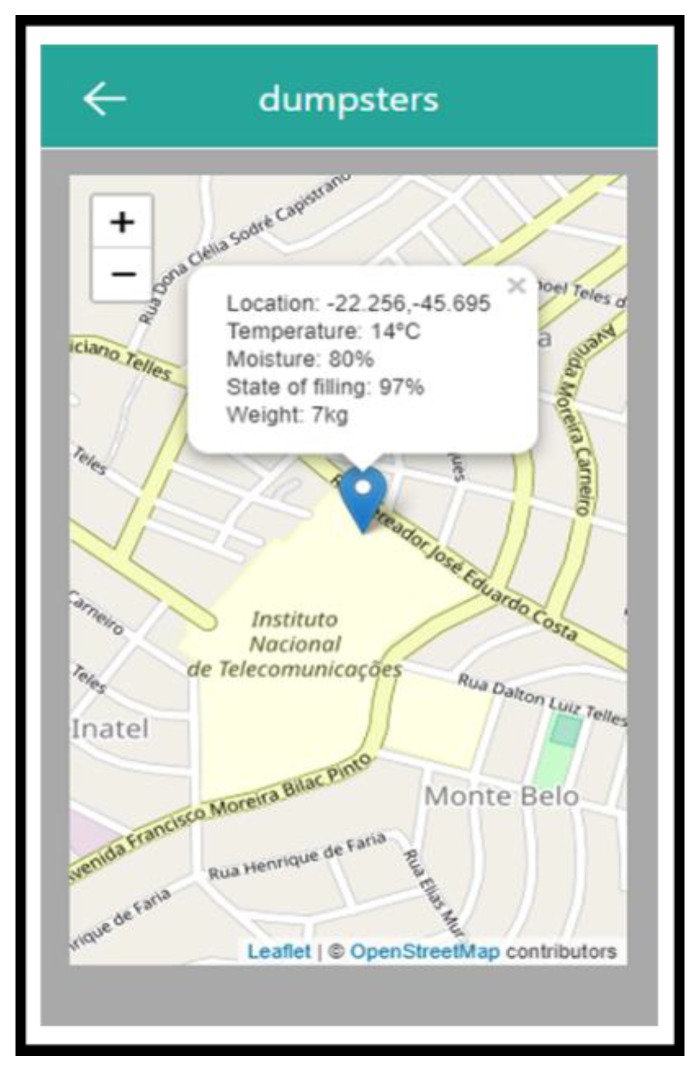
Image of My Waste App.

The system can also be accessed through a Web browser [33], a way for the garbage collection agencies to obtain information on filling of the containers and thus to plan their collection routes in an optimized and pre-scheduled manner, as well as facilitating the insertion of data to be made available to users, such as the collection schedule or out-of-service notifications for maintenance purposes. The system also allows the estimation of places with higher demand for use, which allows the allocation of new collection containers in the areas, with applications responding quickly to the users’ needs. Figure 6 presents a view of My Waste App.

## 4. System Evaluation, Demonstration, and Validation

In this section, the performance evaluation of the proposed system, My Waste Management, is presented through a real-scale experiment use case and corresponding screenshots obtained.

**Figure 7:**
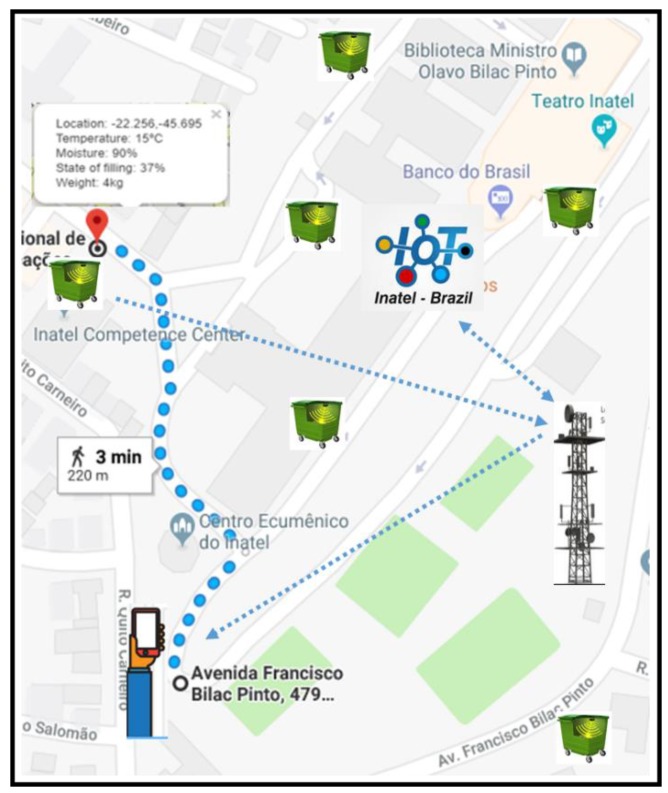
Available route presented by My Waste App.

With the My Waste Bin prototype ready and integrated with the middleware solution for IoT, the In.IoT, it was possible to validate the perfect operation of the solution and guarantee the feasibility of the proposed system. The first step of the experiment was to validate the overall positioning of the My Waste Bin compartment relative to the mobile user using the My Waste App application. The compartment was positioned in an external area, and the geolocation information was transmitted to the In.IoT middleware and later accessed through a smartphone using Android platform. Through the My Waste App application, it was possible to identify the exact positioning of the compartment and to trace the route to its location, as shown in Figure 7.

**Figure 8:**
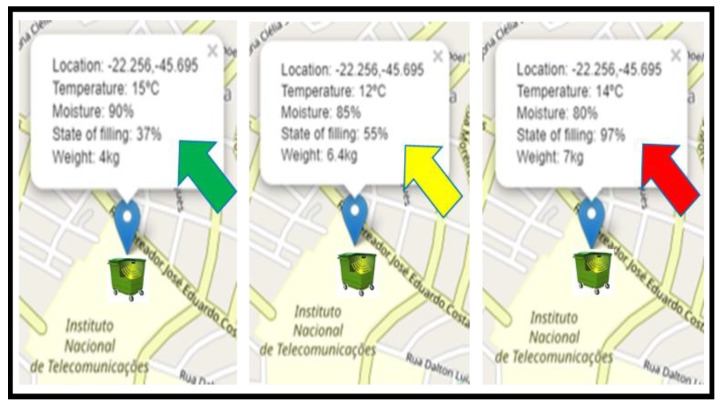
Fill variation presented by My Waste App.

The second part of the experiment was designed to vary the amount of waste deposited inside the compartment and to validate its representation within the application. Such variation alternated the physical characteristics of the residues, from heavier and less voluminous to the inverse situation. Initially, a quantity of residues representing a weight of 4 kg was added to the My Waste Bin compartment, filling 37% of the total capacity of the compartment. Then, additional portions of the same residue were inserted until reaching a weight value equivalent to 6.4 kg, which achieved a filling representation of 55% of the capacity supported by the compartment. Subsequently, another type of residue with different geometry was added to the compartment, in order to make the residue volume a more representative attribute. In this way, the total weight of the residues deposited inside the compartment reached a weight of 7 kg, representing a volume of 97% of the total capacity for the standard of the container chosen during the assembly of the My Waste Bin prototype. The variation in the values mentioned above is shown in Figure 8.

The third and final experiment was the energy source validation of the My Waste Bin. As the prototype has a rechargeable battery system and is powered by a photovoltaic board, the compartment was kept on for a period of 8 weeks, with alternating situations in the solar luminosity presented during the days and did not lose its energy capacity. Within the Arduino Sketch, a timer was considered, with measuring cycles from 5 min, and transmission of information to the In.IoT only happens if the measured values differ from the previous value stored in buffer; other energy saving models are presented in [34,35]. Such an implementation helps to ration the energy of the system. However, during this experimentation phase, a lack of visualization of the battery’s energy level was noticed, something that can be improved in upcoming versions of the system.

Through the above demonstration, it was possible to validate the perfect functioning of the My Waste Bin prototype, which was able to efficiently collect and transmit information on residues deposited in its interior to In.IoT, and, through the application My Waste App collecting information saved in the In.IoT, it was possible to have a perfect interaction with end users.

## 5. Conclusions and Future Work

The society model of the 21st century has been increasingly influenced by cities in their context. According to the United Nations data, by 2050, approximately 70% of the population will live in urban centers, and this rapid growth of people living in cities has been of great concern, since towns do not always grow in a sustainable way. In this regard, smart city design has been increasingly studied and discussed around the world to solve this problem. Following this approach, this paper presented an efficient IoT-based and real-time waste management model for improving the living environment in cities, focused on a citizen perspective. The proposed system uses sensor and communication technologies where waste data is collected from the smart bin, in real-time, and then transmitted to an online platform where citizens can access and check the availability of the compartments scattered around a city.

Taking into account the creation of a real prototype of the smart container and the implementation of a new waste management mobile application and corresponding Web version, and based on the case study experiments, it was concluded that the proposed system can efficiently improve the way people deal with their garbage and optimize economic and material resources.

In future work, the application developed for this solution can be evolved by adding new facilities that can bring to the end user more significant interactions with the management system besides integration with a platform, to calculate the best path in collection routes, seeking efficiency with a lower cost of operating the fleet of trucks. In addition, the investment and operation costs of this solution will be a very interesting study and can be performed as future work.
